# Supplementary material for: MreB filaments align along greatest principal membrane curvature to orient cell wall synthesis
Source: eLife. 2018 Feb 22;7:e32471. doi: 10.7554/eLife.32471 (PMC5854468; doi:10.7554/eLife.32471)
Supplement: Supplementary file 1. [file elife-32471-supp1.docx]

**Table S2 – Strains used in this study**

| Strain | Genotype (all strains are *B. Subtilis* Py79 unless otherwise noted) | Source |
| --- | --- | --- |
| BCW51 | *ycgO::Pxyl-tagO, tagO::erm, amyE::sfGFP-mreB, sinR::phleo* | This work |
| BCW61 | *tagE::erm* | This work |
| BCW72 | *yvhJ::PxylA-mazF (cat)* | This work |
| BCW77 | *ywtF::PxylA-mazF (cat)* | This work |
| BCW78 | *ywtF::msfGFP-ywtF* | This work |
| BCW79 | *yvhJ::msfGFP-yvhJ* | This work |
| BCW80 | *lytR::PxylA-mazF (cat)* | This work |
| BCW81 | *lytR::msfGFP-lytR* | This work |
| BCW82 | *tagO::erm*, *ycgO::PxylA-tagO*, *amyE::Pspac-gfp-mreB (spec)*, *dacA::kan* | This work |
| BDR2061 | *amyE::PxylA-gfp-mbl (spec), mblΩpMUTIN4 (erm)* | (Carballido-López and Errington, 2003) |
| BEB1451 | *hisA1 argC4 metC3 tagO::erm* | (D'Elia et al., 2006) |
| BJS18 | *amyE::PxylA-gfp-mbl (spec)* | (Defeu Soufo and Graumann, 2004) |
| BMD61 | *mbl::mbl-msfGFP (spec)* | This work |
| BRB785 | *yhdG::Pspank-pbpA (phleo)*, *pbpH::spec*, *pbpA::erm*, *mblΩPxylA-gfp-mbl (cat)* | (Garner et al., 2011) |
| BRB786 | *yhdG::PspanK-pbpA (phleo), pbpH::spec, pbpA::cat, yvbJ::PxylA-gfp-mreB (erm)* | (Garner et al., 2011) |
| BEG202 | *∆tagO::erm amyE::Pxyl-gfp-mbl (spec)* | (Schirner et al., 2015) |
| BEG203 | *∆tagO::erm amyE::Pxyl-gfp-mreB (spec)* | (Schirner et al., 2015) |
| BEG281 | *ycgO::PxylA-tagO* | This work |
| BEG291 | *tagO::erm*, *ycgO::PxylA-tagO*, | This work |
| BEG275 | *amyE::Pspac-gfp-mreB (spec)* | (Meeske et al., 2016) |
| BEG300 | *tagO::erm, ycgO::PxylA-tagO, amyE::Pspac-gfp-mreB (spec),* | This work |
| BRB4282 | *168 trpC2 ΔtagO::erm* | (D'Elia et al., 2006) |
| bAB343 | *ftsZ::mNeonGreen-15aa-ftsZ, amyE::spc-Pspank-mciZ, ycgO::cat-Pxyl-tagO, tagO::erm* | This work |
| bAB327 | *ftsZ::mNeonGreen-15aa-ftsZ, amyE::Phyperspank-minCD, ycgO::Pxyl-tagO, tagO::erm* | This work |
| bAB388 | *ftsZ::mNeonGreen-15aa-ftsZ, amyE::Physpank-ftsA ycgO::cat-Pxyl-tagO, tagO::erm* | This work |
| bYS09 | *mreB::mreB-40aa-mNeonGreen* | This work |
| bYS40 | *mreB::HaloTag–MreB* | This work |
| bYS201 | *HaloTag-Pbp2A::cat* | This work |
| bAB198 | *tagT::erm-Pxyl-HaloTag-15aa-tagT* | This work |
| bAB197 | *tagV::erm-Pxyl-HaloTag-15aa-tagV* | This work |
| bAB196 | *tagU::erm-Pxyl-HaloTag-15aa-tagU]* | This work |
| NO50 | *E. coli - msfGFP-MreB(sw)* | (Ouzounov et al., 2016) |
| RM478 | *E. coli - ΔrodZ (cam), mreBS14A-msfGFPSW (kan)* | (Morgenstein et al., 2015) |
