# Supplementary material for: MreB filaments align along greatest principal membrane curvature to orient cell wall synthesis
Source: eLife. 2018 Feb 22;7:e32471. doi: 10.7554/eLife.32471 (PMC5854468; doi:10.7554/eLife.32471)
Supplement: Supplementary file 2. [file elife-32471-supp2.docx]

**Table S3 – Oligonucleotides used in this study**

| Primer | Sequence |
| --- | --- |
| oCW054 | TGCAATTTCAGGGTTGACTG |
| oCW055 | ATACGAACGGTACTGAGCGAGGGAGCAGAACGGCATCTAGAATATATGATCATTG |
| oCW056 | ACTTATTAAATAATTTATAGCTATTGAAAAGAGAT |
| oCW057 | TTTTCAATAGCTATAAATTATTTAATAAGTCTTGGAGGGTCACGGAAATAAA |
| oCW058 | TTTCATCCTTGTTTTCAGGCTA |
| oCW072 | ATGCGAAAAGGGGAAGAATTGTTTA |
| oCW073 | GCCGCTTCCTTGGCCTGA |
| oCW100 | TCCGTATGGAGATGGAGAGG |
| oCW101 | CCGCTTATCCTTTTCACAGC |
| oCW109 | ATACGAACGGTAGTTGACCAGTGCTCCCTGCCTTTGCACCTCGTCTGTTAAAT |
| oCW125 | AAAATTAACGTACTGATTGGGTAGTCTAGAATGAGAAACGAACGCAGAAAAAAG |
| oCW137 | GCCTGTAAACAATTCTTCCCCTTTTCGCATCCTTTGCACCTCGTCTGTTAAAT |
| oCW138 | CAGGGACCGGGCTCAGGCCAAGGAAGCGGCATGAGAAACGAACGCAGAAAAAAG |
| oCW139 | ATAAACGGTTTCTCGCATGG |
| oCW140 | GCAGCTTATGGGCTTTTTCA |
| oCW141 | ATACGAACGGTAGTTGACCAGTGCTCCCTGTTATTCAGTCTCCTTTATGTGATTGA |
| oCW142 | AAAATTAACGTACTGATTGGGTAGTCTAGAATGGCTGAACGCGTTAGAGTG |
| oCW143 | GAGCTGTCAGTCCCGTCTTC |
| oCW144 | TTGTTTGAGCTTGTGCTGCT |
| oCW145 | CAGGGACCGGGCTCAGGCCAAGGAAGCGGCATGGCTGAACGCGTTAGAGTG |
| oCW146 | GCCTGTAAACAATTCTTCCCCTTTTCGCATTATTCAGTCTCCTTTATGTGATTGAC |
| oCW155 | GAGCAATGGCTAAAGCGTCT |
| oCW156 | GCAGGTACGGATGAGGAAAA |
| oCW159 | ATACGAACGGTAGTTGACCAGTGCTCCCTGATATCAATACCTCACGTTTCTTTAATATTT |
| oCW160 | GCCTGTAAACAATTCTTCCCCTTTTCGCATATATCAATACCTCACGTTTCTTTAATATTT |
| oCW161 | CTTTGCTTTCTTCGCCATTC |
| oCW162 | GGATCGAACAGCTCCTTCAG |
| oCW163 | CAGGGACCGGGCTCAGGCCAAGGAAGCGGCATGGAAGAACGATCACAGCGC |
| oCW164 | AAAATTAACGTACTGATTGGGTAGTCTAGAATGGAAGAACGATCACAGCGC |
| oCW165 | CGCCATCCCGTTCATTATAC |
| oCW166 | TCAGCTTGCCTGTGAAACAT |
| oMD44 | AATTCTCGAAGGAGAGCCTGTTC |
| oMD47 | TGATTTCACAAACCTCATTCTGAAAAAGAATGAGGTTTTTTTATGAAAAATTCTGCTCCCTCGCTCAG |
| oMD48 | CGTCATTTAACATCTTTTCGTGAAGGCCAGGGAGCACTGGTCAAC |
| oMD50 | CGTGAACTCATCATCGCTCC |
| oMD56 | TTCTTTTTCAGAATGAGGTTTGTGAAATCATTTGTAAAGTTCATCCATTCCATGCG |
| oMD069 | CTGGACAGGGCTCAGGTCCGGGATCTGGCATGAGGAGAAATAAACCAAAAAAGCAAAATC |
| oMD082 | TAATCGCTCCAGTTTGAAGACCG |
| oMD083 | AGCAGTCAGGGAAATTTATTGATTTGG |
| oMD90 | TGGCCAGGGACCGGGCTCAGGCCAAGGAAGCGGCATGCGAAAAGGGGAAGAATTGTTTAC |
| oMD092 | TAACGGGAGTTCAATAGAAGAGGTG |
| oMD116 | CAGCCAGAACTGCTATCAATATCACTAC |
| oMD134 | CCGAGCAGGGAAGATATTGAAG |
| oMD262 | GCCTTGACCTGGGCCAGATC |
| oMD108 | ACGAACGGTAGTTGACCAGTGCTCCCTGTCTTGACACTCCTTATTTGATTTTTTGAAGAC |
| oMD191 | TTTGGATGGATTCAGCCCGATTG |
| oMD196 | GGGCAAGGCTAGACGGG |
| oMD197 | TCACATACTCGTTTCCAAACGGATC |
| oMD234 | ATACGAACGGTACTGAGCGAGGGAGCAGAATAATGGATTTCCTTACGCGAAATACG |
| oEG85 | GAGAGCTTGATGTCACAAGCAGCTGGGAAGGAATTCGTGCCATGTCACTATTGCT |
| oEG86 | ATAAAGAAGTCTCCTTTGGACTCGAGGCATTCAAATACAGATGCATTTTATTTCATA |
| oEG87 | TGAAATAAAATGCATCTGTATTTGAATGCCTCGAGTCCAAAGGAGACTTCTTTATGCTTG |
| oEG88 | AATAAGGGTAACTATTGCCGTATGGGATCCATGCTAGCTTAATTCCTTTTCACCAGCCG |
| oJM028 | TTCTGCTCCCTCGCTCAG |
| oJM029 | CAGGGAGCACTGGTCAAC |
| oMK047 | TCTAGACTACCCAATCAGTACGTT |
| oYS007 | CCTGGACAAGGATCTGGCCCAGGTCAAGGCATGGTTTCGAAAGGAGAGGAGG |
| oYS008 | GTTACACCTCTTCTATTGAACTCCCGATTACTTATAGAGTTCATCCATACCCATCACG |
| oYS136 | AACATCACCTTTTCTATTTACAGAAAGAAAAGACA |
| oYS598 | GCCAGATCCCGGACCTGAGCCCTGTCCAGAGCCGCCGCTGATTTCTAAGGTAGAAAG |
| oYS599 | GAGTGTCTTTTCTTTCTGTAAATAGAAAAGGTGATGTTATGGCAGAAATCGGTACTGGC |
| oYS602 | GCCGCTGCCTTGTCCA |
| oYS603 | CAAGGCCCTGGATCTGGACAAGGCAGCGGCATGGCAGAAATCGGTACTGGC |
| oYS604 | GTTACACCTCTTCTATTGAACTCCCGATTAGCCGCTGATTTCTAAGGTAGAAAG |
| oSW76 | TAGATCACCTCCTTAAGCTT |
| oAB291 | CGGTAAGTCCCGTCTAGCCTTGCCCTTATGGCTTTGAGATCCAATCTTT |
| oAB307 | CAATTAAGCTTAAGGAGGTGATCTAGTGAAAGTGCACCGCATGCC |
| Sinr_up_F | CAGTTGAAATGGACAAACAAATC |
| Sinr_up_R | ACTGAGCGAGGGAGCAGAAGTGTCATCACCTTCCTTG |
| Sinr_DOWN_F | GTTGACCAGTGCTCCCTGTGCCTGAGCAGAGGC |
| Sinr_DOWN_R | GGACAGCACCATGTCTACTTAAC |
