# Supplementary material for: MreB filaments align along greatest principal membrane curvature to orient cell wall synthesis
Source: eLife. 2018 Feb 22;7:e32471. doi: 10.7554/eLife.32471 (PMC5854468; doi:10.7554/eLife.32471)
Supplement: Supplementary file 3. — Related to Figure 4 and Appendix 1. [file elife-32471-supp3.docx]

**Table S1: Model Parameters. Related to Figure 4 and Supplemental Text 1.**

| **Quantity** | **Estimate** | **Source** |
| --- | --- | --- |
| **MreB values** |  |  |
| MreB bound length $l_{b}$ | 220 nm | This work |
| MreB monomer length $l_{MreB}$ | 51 angstroms | (van den Ent et al., 2014) |
| MreB cross-sectional radius $r_{MreB}$ | 3.2 nm | (van den Ent et al., 2014) |
| MreB wild-type principal radius of curvature $R_{MreB}$ | 300 nm | This work |
| MreB Young’s modulus $Y_{MreB}$ | Similar to actin; 2 GPa | (1994) |
| MreB cross-sectional binding fraction $b$ | 0 | This work |
| **Cell values** |  |  |
| *B. subtilis periplasm thickness* $h_{peri}$ | 22 nm | (Matias and Beveridge, 2005) |
| *B. subtilis* cross-sectional radius $R_{cell}$ | 500 nm | This work |
| *B. subtilis* internal osmotic pressure $p_{cell}$ | 20 atm | (1990) |
| **Binding energy values** |  |  |
| Unit MreB-cell membrane interaction energy $E_{int}^{0}$ | 10 kT | This work |
| Absolute temperature $T$ | 300 K | This work |
| *B. subtilis* typical cross-sectional radius $R_{cell}^{*}$ for losing shape | about 1-1.5 microns | This work |
